# dbCRAF: a curated knowledgebase for regulation of radiation response in human cancer

**DOI:** 10.1093/narcan/zcae008

**Published:** 2024-02-24

**Authors:** Jie Liu, Jing Li, Fangfang Jin, Qian Li, Guoping Zhao, Lijun Wu, Xiaoyan Li, Junfeng Xia, Na Cheng

**Affiliations:** Key Laboratory of Intelligent Computing and Signal Processing of Ministry of Education and Information Materials and Intelligent Sensing Laboratory of Anhui Province, Institutes of Physical Science and Information Technology, Anhui University, Hefei, Anhui 230601, China; Key Laboratory of Intelligent Computing and Signal Processing of Ministry of Education and Information Materials and Intelligent Sensing Laboratory of Anhui Province, Institutes of Physical Science and Information Technology, Anhui University, Hefei, Anhui 230601, China; Key Laboratory of Intelligent Computing and Signal Processing of Ministry of Education and Information Materials and Intelligent Sensing Laboratory of Anhui Province, Institutes of Physical Science and Information Technology, Anhui University, Hefei, Anhui 230601, China; School of Environmental Science and Optoelectronic Technology, University of Science and Technology of China, Hefei, Anhui 230026, China; Key Laboratory of High Magnetic Field and Ion Beam Physical Biology, Hefei Institutes of Physical Science, Chinese Academy of Sciences, Hefei, Anhui 230031, China; Key Laboratory of Intelligent Computing and Signal Processing of Ministry of Education and Information Materials and Intelligent Sensing Laboratory of Anhui Province, Institutes of Physical Science and Information Technology, Anhui University, Hefei, Anhui 230601, China; Key Laboratory of High Magnetic Field and Ion Beam Physical Biology, Hefei Institutes of Physical Science, Chinese Academy of Sciences, Hefei, Anhui 230031, China; Key Laboratory of Intelligent Computing and Signal Processing of Ministry of Education and Information Materials and Intelligent Sensing Laboratory of Anhui Province, Institutes of Physical Science and Information Technology, Anhui University, Hefei, Anhui 230601, China; Key Laboratory of Intelligent Computing and Signal Processing of Ministry of Education and Information Materials and Intelligent Sensing Laboratory of Anhui Province, Institutes of Physical Science and Information Technology, Anhui University, Hefei, Anhui 230601, China; School of Biomedical Engineering, Anhui Medical University, Hefei, Anhui 230032, China

## Abstract

Radiation therapy (RT) is one of the primary treatment modalities of cancer, with 40–60% of cancer patients benefiting from RT during their treatment course. The intrinsic radiosensitivity or acquired radioresistance of tumor cells would affect the response to RT and clinical outcomes in patients. Thus, mining the regulatory mechanisms in tumor radiosensitivity or radioresistance that have been verified by biological experiments and computational analysis methods will enhance the overall understanding of RT. Here, we describe a comprehensive database dbCRAF (http://dbCRAF.xialab.info/) to document and annotate the factors (1,677 genes, 49 proteins and 612 radiosensitizers) linked with radiation response, including radiosensitivity, radioresistance in cancer cells and prognosis in cancer patients receiving RT. On the one hand, dbCRAF enables researchers to directly access knowledge for regulation of radiation response in human cancer buried in the vast literature. On the other hand, dbCRAF provides four flexible modules to analyze and visualize the functional relationship between these factors and clinical outcome, KEGG pathway and target genes. In conclusion, dbCRAF serves as a valuable resource for elucidating the regulatory mechanisms of radiation response in human cancers as well as for the improvement of RT options.

## Graphical Abstract

**Graphical Abstract:**
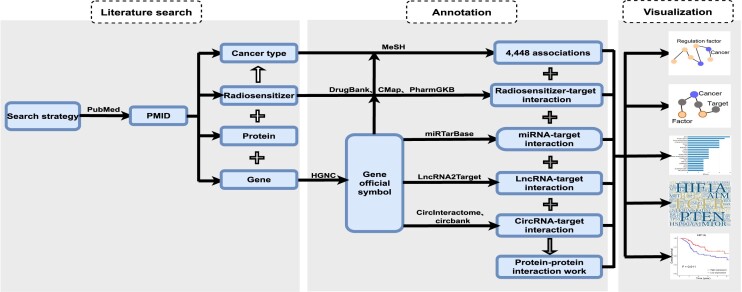

## Introduction

As an intricate disease, cancer has greatly reduced human life expectancy worldwide (1,2). Radiation therapy (RT) is one of the prominent modalities for treating intricate diseases, and it can be used alone or in combination with other treatments to cure tumors and relieve tumor-related symptoms in cancer patients (3). However, the effects of RT are not specific to tumor cells and may cause side effects or toxicity to surrounding exposed organs and tissues (e.g. RT injury, secondary cancer) (3–5). Therefore, it is important to examine the molecular mechanism of radiation response that enhances tumor killing while reducing toxicity to surrounding normal tissues, which can ensure better therapeutic outcomes and provide meaningful improvement for cancer patients (6–8).

Many researchers have dedicated their efforts to studying the molecular mechanisms of radiosensitivity, radioresistance in human tumor cell lines or prognosis in cancer patients receiving RT, but these valuable findings are scattered across multiple sources (9–11). To be specific, emerging evidence in the literature has connected more coding genes and various types of non-coding RNAs (ncRNAs) like microRNAs (miRNAs), long non-coding RNAs (lncRNAs) and circular RNAs (circRNAs) to radioresistance and radiosensitivity in human cancers. For example, Han *et al.* demonstrated that down-regulation of *PVT1* inhibits radioresistance in nasopharyngeal carcinoma cells (12). Wang et al. found that circ_0067835 knockdown suppresses colorectal cancer (CRC) progression and strengthens CRC cell radiosensitivity (13). Additionally, Wen *et al.* constructed the dbCRSR database, which contains 395 coding genes, 119 miRNAs and 306 chemical compounds that can modulate radiosensitivity (14). Moreover, with the development of sequencing technology, there has been extensive literature using computational analysis methods to elucidate the complex relationship between genes and cancer prognosis with RT (15–19). These findings also provide valuable insights for improving the clinical outcomes of RT. To sum up, the lack of an effective collection of these scattered data makes it difficult to obtain an overarching understanding of prior research on the molecular mechanisms of radiation response, thus hampering the further progress of RT treatment.

To meet these needs, we developed an informative and functional database (dbCRAF, http://dbCRAF.xialab.info/), which contains various factors (1,677 genes, 612 radiosensitizers and 49 proteins) associated with radiosensitivity, radioresistance in cancer cells and prognosis in cancer patients receiving RT. In addition, we collated miRNA-target, lncRNA-target, circRNA-target and drug-target interactions for ncRNAs and radiosensitizers associated with radiation response, allowing users to construct radiation response interaction networks. Furthermore, four analytics modules were designed to enable users to extract key factors or potential modulators affecting radiation response based on the collected data. In brief, dbCRAF aspires to be a one-stop online service for integrating and analyzing valuable information on cellular radiation response.

## Materials and methods

This part introduces data collection and curation, interaction data processing, data analysis and visualization, and database design and web interface implementation for the dbCRAF database.

### Data collection and curation

To conduct a systematic review of the literature, we first searched PubMed articles published between January 2000 to February 2022 using a list of keywords (Supplementary Table S1) related to radiosensitivity, radioresistance and RT prognosis prediction. Second, we performed full-text screening to assess the eligibility of papers and extracted key information from the relevant articles using PubTator (20), including publication information, the factor (genes, proteins, radiosensitizers) that could regulate radiation response, cancer type, the regulation mode of each factor, gene expression fold change, the cytotoxicity of radiosensitizers, the cell line name, the type of model organism, the type of ray, the cancer tissue source, sample size, collection time, the type of radiotherapy and survival endpoint. Notably, we defined each evidence that linked a factor to radiation response in cancer as an association. Third, to guarantee consistent names for all obtained factors and related cancers, we unified the gene names using the gene symbol-alias conversion table from the HGNC database (2022.4.12 version) (21), and radiosensitizer using ‘Generic Name’ from the DrugBank database (22). Fourth, we harmonized cancer names with disease terms in the MeSH database (2022.8.8 version) according to the occurrence location of the tumor (23). Fifth, we retained the original content of the type of RT, cancer tissue source, the type of ray and survival endpoint due to the presence of multiple descriptions in many publications. This decision enables the database to provide a more comprehensive representation of the data. Lastly, we added the subcellular location annotation from the GeneCards database, exploring the relationship between different subcellular structures and gene products related to radiation response (24).

All the above information has been processed using python or R scripts, and manually verified by at least two individuals to ensure its accuracy. The scripts employed for processing could be downloaded in dbCRAF.

### Interaction data processing

To enable users to mine more information based on the associations collected in dbCRAF and to comprehend the mechanisms underlying radiation response regulation, we gathered four kinds of interaction data and target genes of miRNAs, lncRNAs, circRNAs, and radiosensitizers related to radiation response, as shown in Supplementary Table S2. We first collected the miRNA-target interactions of Homo sapiens with the support type of ‘Functional miRNA-target interactions’ from the miRTarBase database (25). Then, we obtained the lncRNA-target interactions in human that were confirmed by low-throughput methods such as RT-qPCR and western blot from LncRNA2Target database (26). Next, we organized the intersection of potential circRNA-target interactions from the CircInteractome (27) and Circbank (28) databases to improve confidence of these results derived from related predictive tools. Moreover, radiosensitizer (drug)–target interactions were retrieved from the DrugBank, PharmGKB (29) and CMap (30) databases. Finally, we also integrated protein-protein interaction (PPI) data for the target genes and the genes recorded in dbCRAF based on the STRING database (31) to infer the overall regulatory network.

### Data analysis and visualization

To further explore the association data and four kinds of interaction data, we constructed four analysis blocks equipped with visual aids such as Word Cloud graphs, Kaplan–Meier survival analysis, Kyoto Encyclopedia of Genes and Genomes (KEGG) pathway enrichment analysis (32) and interaction network visualization. First, Word Cloud allows users to count and visualize the associations between factors and cancer recorded in dbCRAF. The size of each factor's representation in the Word Cloud is proportional to the number of associations it has. This visual representation helps users identify factors that have a higher number of associations. Second, we collected the RNA-seq and clinical data from the Cancer Genome Atlas (TCGA) database (33), and investigated whether the expression levels of miRNAs or mRNAs recorded in dbCRAF affected the clinical prognosis of samples receiving RT at different tumor stages (stages I–IV) in one or more types of cancer. The results are presented using Kaplan-Meier survival curves, enabling users to assess the impact of these factors on patient outcomes. Third, KEGG pathway enrichment analysis supports users to annotate genes related to radiation response according to the type of pathway involved, and the results are presented in the form of bubble chart. This visualization helps users understand the functional roles of the identified factors by displaying enriched pathways. Finally, interaction network visualization enables users to describe the interaction relationships of factors in dbCRAF, thus facilitating the screening of key genes and potential modulators of radiation response.

### Database design and web interface implementation

The dbCRAF website was developed using Apache Tomcat Server (http://tomcat.apache.org), Flask (https://pypi.org/project/Flask) and MySQL (http://www.mysql.org). We used Python 3.10 for server-side scripting to provide query and computation support in the backend of the database, and used HTML5, CSS3, JQuery (version 1.12.4), AJAX, ECharts (https://echarts.apache.org), dataTables (https://datatables.net) and BootStrap (https://getbootstrap.com) frameworks to develop a user-friendly interactive web interface. Additionally, we employed various Python packages for the implementation of the ‘Analysis’ interface, including lifelines (version 0.27.0), gseapy (version 0.10.8), matplotlib (version 3.5.2), numpy (version 1.22.4), pandas (version 1.4.2) and matplotlib (version 3.5.2). We recommend visiting dbCRAF by a web browser such as Google Chrome, Microsoft Edge or Firefox.

## Results

### Database content summary

After excluding duplicate articles with the same PubMed ID or title, we retained >10,000 records. Particularly, we classifies those literature about exploring the molecular mechanism of radiosensitivity and radioresistance through over-expression or knock-down assays as ‘Experiment’, and labels those about predicting radiation response and RT prognosis of cancer patients through computational analysis as ‘Prediction’. We then screened the full-text records to assess their relevance and extracted key information. dbCRAF currently contains 4,448 association entries between factors and cancer from 2,670 publications and it covers 1,677 genes, 612 radiosensitizers, and 49 proteins linked with 30 types of cancer.

In comparison to the dbCRSR database, the dbCRAF database encompassed literature with a longer time span, as indicated in Table 1. Additionally, dbCRAF provided information on multiple mechanisms of radiation response, resulting in a greater number of associations between factors and radiation response in cancer being recorded. Moreover, dbCRAF included descriptions of the various modes through which each factor modulated the radiation response in cancer. These modes included the addition of radiosensitizers, occurrence of gene mutation, changes in gene expression level, gene methylation status or protein expression. In summary, the information contained in dbCRAF is more comprehensive.

**Table 1.** The statistic and comparison of dbCRSR and dbCRAF databases

Term	dbCRSR	dbCRAF
Database function	Download, search	Download, search, analysis
Publication source	PubMed	PubMed
Publication date	January 2008–October 2017	January 2000–February 2022
Publication number	1,072	2,670
Association number	1,132	4,448
Mechanism	Experiment (RS^a^)	Experiment (RS/RR^b^), prediction
Factor type	mRNA (395), miRNA (119), compound (306)	Gene (1,677), protein (49), radiosensitizer (612)

^a^Radiosensitivity.

^b^Radioresistance.

**Figure 1.**
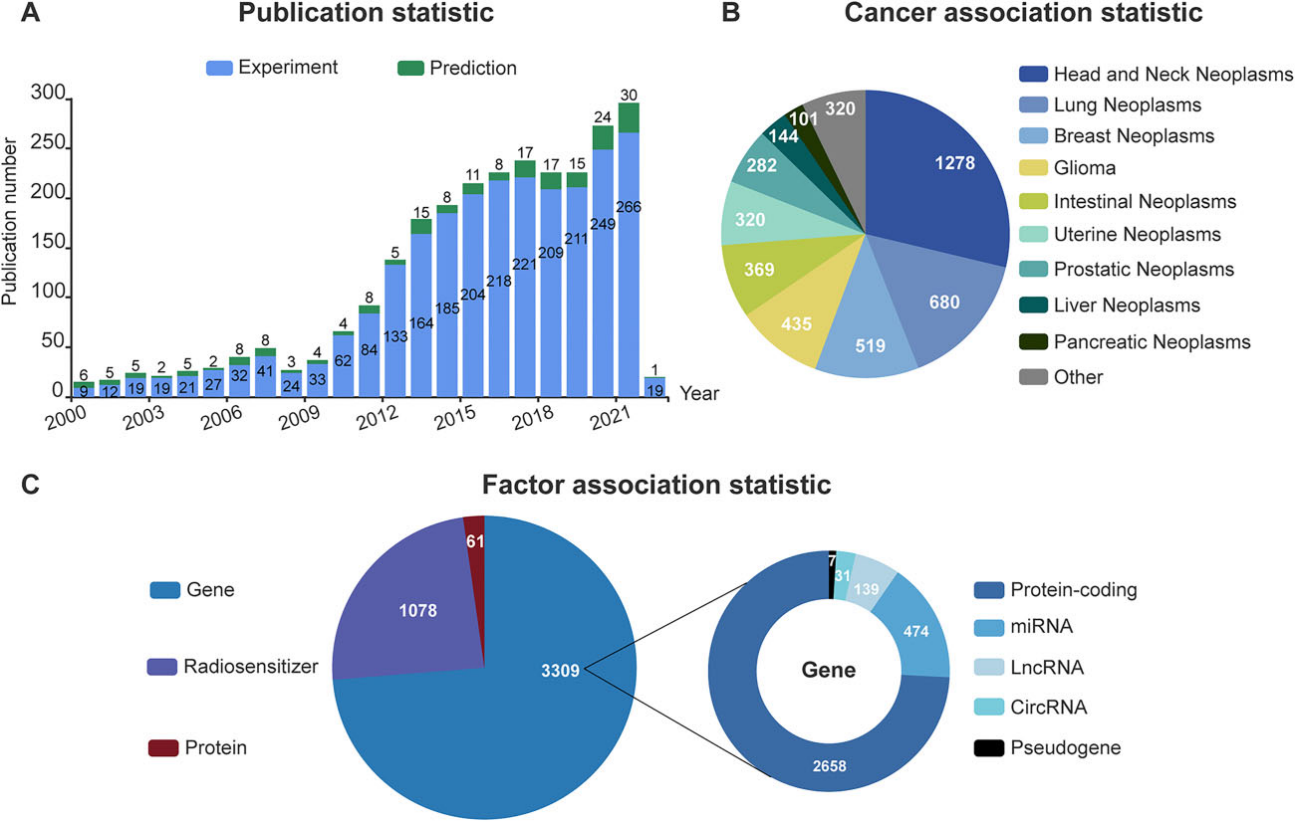
Summary of dbCRAF contents. (**A**) Statistics of publication information. (**B**) Statistics of association data with each cancer type. (**C**) Statistics of association data with each kind of factor.

In addition, we made three findings by analyzing the 4,448 associations recorded in dbCRAF. First, the number of studies that focused on molecular mechanisms of radiation response showed an increasing trend year by year (Figure 1A). This indicates the growing interest in understanding the molecular mechanisms of radiation response and its potential applications in cancer treatment. Meanwhile, recent studies published since 2018 have integrated the analysis of large genomic or transcriptome datasets with biological experiments to efficiently screen and verify genes of interest. This approach eliminated the limitations imposed by prior knowledge or assumptions and provided stronger evidence for identifying potential new associations between factors and cancer. Second, more than half of the associations were related to head and neck neoplasms, lung neoplasms, and breast neoplasms (Figure 1B), which might be due to their higher incidence or the wider use of RT options and thus leading to more relevant studies (1,34–36). Third, the largest number of factors involved in regulating cancer radiation response were genes, followed by radiosensitizers (Figure 1C). Lastly, we separately counted the five categories of genes and found that in addition to protein-coding genes, there has been an increasing amount of ncRNAs documented in the radiation response-related literature (37,38).

### Hot protein-coding gene and enriched pathways

**Figure 2.**
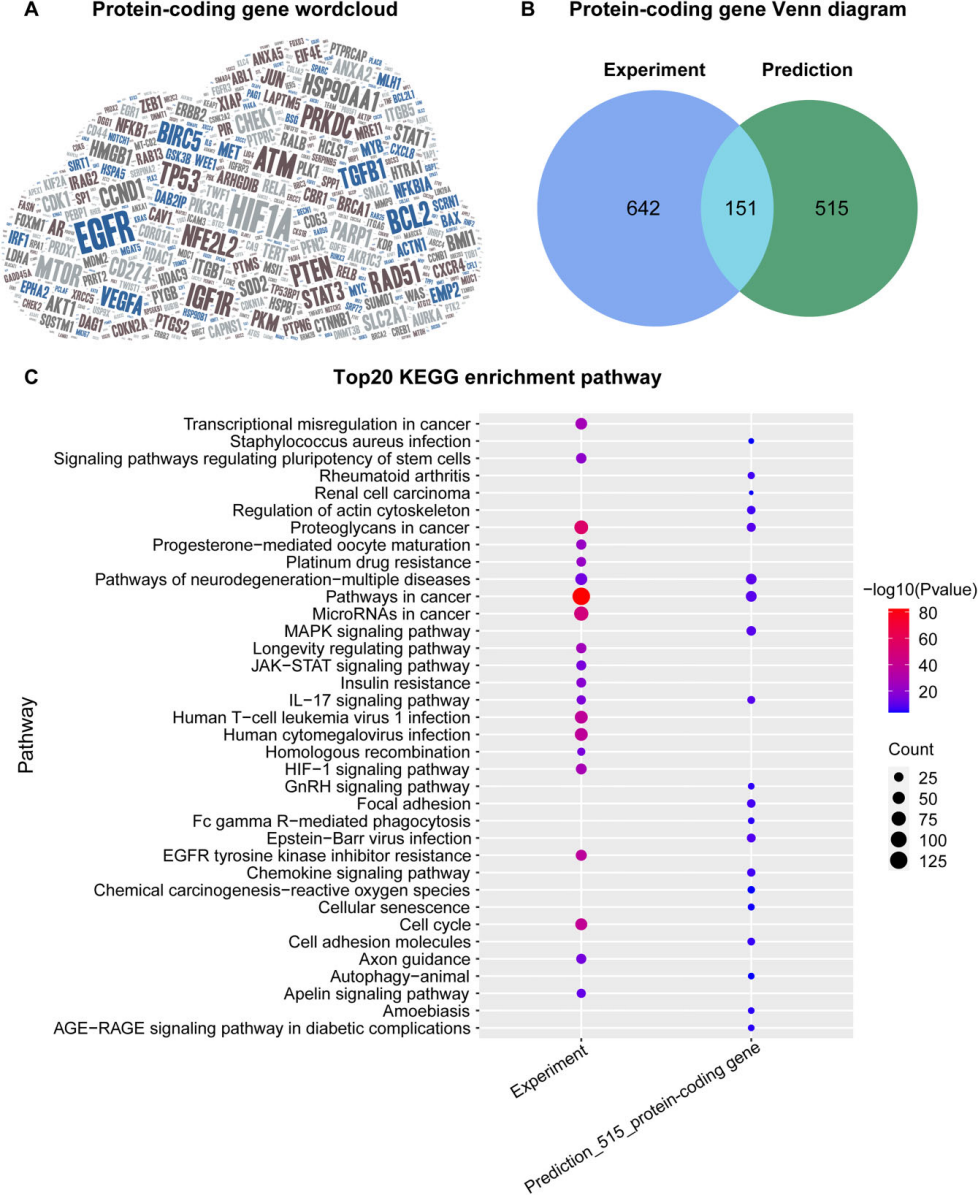
Overview of protein-coding gene-cancer associations. (**A**) Word cloud of all protein-coding genes, where there is more literature evidence for genes are displayed using a larger font size. (**B**) Number of coding genes derived from Experiment or Prediction publications. (**C**) The top 20 KEGG enrichment pathways of coding genes with experimental evidence (793 genes) or only computational analysis results (515 genes).

After counting 2,658 associations between coding gene and cancer, we found a total of 1,308 coding genes. Among them, *HIF1A*, *EGFR*, and *ATM* were the most extensively studied coding genes, and their expression levels could influence the response of multiple tumors to radiation (Figure 2A). Next, we grouped all coding genes (793 versus 666) collected in ‘Experiment’ and ‘Prediction’ articles respectively, and obtained 151 genes shared between them (Figure 2B). This indicates that both biological experiments and computational methods have identified these 151 genes as potential genetic biomarkers for prognosis, radiosensitivity or radioresistance in human cancer. This finding suggests the possibility of optimizing RT effects based on a patient's genomic profiles and intrinsic radiosensitivity (39).

Additionally, we analyzed 515 coding genes related to cancer prognosis identified in the ‘Prediction’ articles but not in the ‘Experiment’ articles to uncover more potential genetic biomarkers. Specifically, we performed the KEGG pathway enrichment analysis on the two groups of coding genes (793 versus 515) with or without experimental evidence of regulating radiation response. The results showed that four pathways were the same in both groups of genes, including IL-17 signaling pathway, neurodegeneration-multiple diseases pathways, pathways in cancer, and proteoglycans in cancer (Figure 2C). In summary, 137 of the 515 coding genes are involved in one or more of these four pathways (Supplementary Table S3), and it is likely that they regulate radiosensitivity, radioresistance or prognosis in cancer through their involvement in these functional pathways.

### Noncoding RNA, radiosensitizer and their target genes analysis

**Figure 3.**
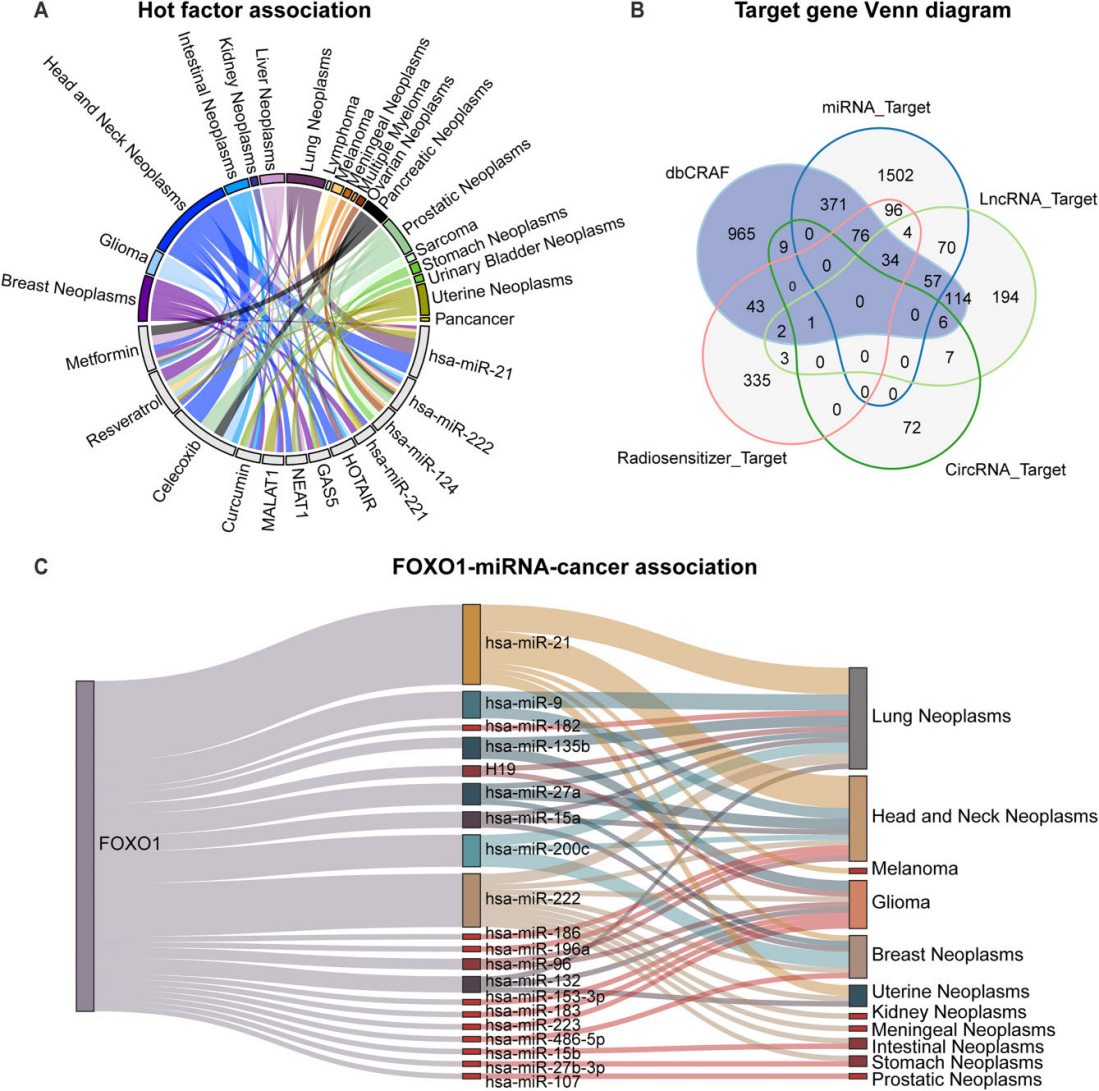
Statistics related to non-coding RNAs, radiosensitizers and their target interactions. (**A**) Statistics of the associations about top four miRNAs, lncRNAs and radiosensitizers, where more frequent associations are displayed using a thicker line. (**B**) Venn diagram of genes recorded in dbCRAF and all target genes. (**C**) Sankey diagram of FOXO1–miRNA–cancer associations.

Non-coding RNAs and radiosensitizers typically exert regulatory function in radiation response via interacting with target genes. Therefore, we collected and analyzed interaction data of ncRNAs and radiosensitizers related to radiation response from various databases (Supplementary Table S2). First, we counted the most studied miRNAs, lncRNAs and radiosensitizers based on association data documented in dbCRAF. As shown in Figure 3A, the top four ncRNAs and radiosensitizers were all involved in modulating radiation response in one or more cancers. Notably, there were only 31 associations between circRNA and cancer, making it difficult to sort circRNAs according to the study frequency. Second, we integrated all target genes and interaction data of ncRNAs and radiosensitizers linked with radiation response, including miRNA-target interactions, lncRNA-target interactions, circRNA–target interactions and radiosensitizer-target interactions. Interestingly, almost 25% of the target genes have been documented to be associated with radiation response (Figure 3B). Third, we counted the top 20 target genes that appeared most frequently in the interaction data and collected evidence for their regulation of radiation response. We hypothesized that target genes that appear more frequently in the interaction data are more likely to regulate radiosensitivity or radioresistance. Coincidentally, 19 of the top 20 target genes have been experimentally confirmed to regulate the radiation response (Supplementary Figure S1), with only the *FOXO1* gene lacking study evidence.

Therefore, we combined miRNA-target (*FOXO1*) interactions and relevant associations between miRNA and cancer recorded in dbCRAF to map *FOXO1*-miRNA-cancer associations. The results showed that *FOXO1* may be associated with radiation responses in lung neoplasms and head and neck neoplasms (Figure 3C). Although there is no direct evidence suggesting that *FOXO1* regulates the radiation response of cancer cells, *FOXO1* is still an important factor in cancer processes based on its inclusion in the oncogene and tumor suppressor gene list from the Cancer Gene Census project (40). Additionally, Zhou *et al.* found that *TCF19* could promote cell proliferation in non‐small cell lung cancer by inhibiting *FOXO1* (41). Gong *et al.* also showed that the *AKBA* gene inhibited radioresistance in lung cancer through the regulation of the *AKT*/*FOXO1*/*p21* axis (42). These findings support our conclusion that *FOXO1* may be closely related to the occurrence and prognosis of RT in lung cancer.

### Key dbCRAF modules and user cases

**Figure 4.**
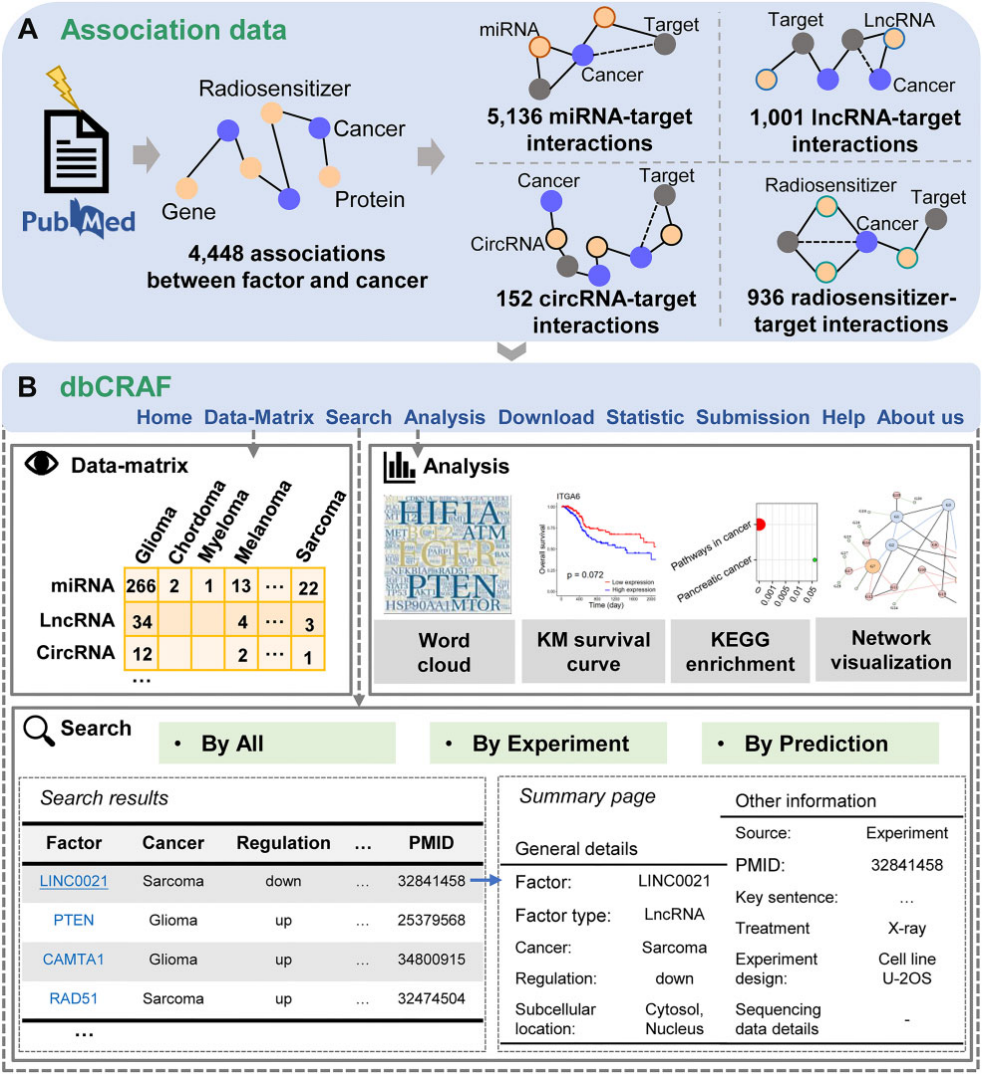
Schematic of dbCRAF.

dbCRAF provides a comprehensive collection of high-quality data, including association data between factors and radiation response in cancer as well as interaction data between factors and target genes. All data are freely available through the Download module, and users can browse and analyze the data using the Data-Matrix, Search, and Analysis modules on the dbCRAF website (http://dbCRAF.xialab.info/), as shown in Figure 4.

The ‘Data-Matrix’ page is an interactive and digitized table organized by factors (rows) associated with radiation response and cancer types (columns), allowing users to quickly navigate all association entries.

For the Search page, dbCRAF provides user-friendly search options supporting automatic retrieval of association data recorded in dbCRAF under multiple cancer types. In addition, users can query the related data with different evidence in three ways: ‘By All’, ‘By Experiment’ and ‘By Prediction’. Furthermore, users can click the corresponding entry to view all detailed information, such as key sentences and biological experiment settings.

On the Analysis page, dbCRAF supports four practical analysis functions to answer common biological questions. Users can perform word cloud, KEGG pathway enrichment analysis, and interaction network visualization using a list of factors related to radiation response along with their interaction data. Kaplan-Meier survival analysis is implemented with the miRNA-seq, mRNA-seq and clinical data of cancer patients treated with RT in the TCGA database.

## Discussion

To the best of our knowledge, dbCRAF is the first database to furnish association data between factor and human cancer related to multiple radiation response regulatory mechanisms, including radiosensitivity, radioresistance, and prognosis of RT in cancer patients. At the same time, we gathered the interaction data concerning ncRNAs and radiosensitizers linked to radiation response. On top of that, dbCRAF allows users to analyze the above information, and provides an all-in-one service for the retrieval, analysis, and visualization of human cancer radiation response factors related to human cancer.

For this study, we ran statistical analyses of association data retrieved from the PubMed literature, interaction data and target genes derived from multiple databases, respectively, to provide more guidance for researchers. Moreover, we found that out of the 666 coding genes related to tumor RT prognosis, a total of 151 genes had been validated by over-expression or knock-down assays. These assays have demonstrated their potential influence on the radiosensitivity or radioresistance of cancer cell lines. Therefore, it is essential to perform biological experiments on the remaining 515 genes to investigate their regulatory mechanism of response to radiation in human cancer cells. Additionally, regarding the data of miRNA-target interactions, we observed that hsa-miR-195–5p interacted with *BCL2*, the target gene with the most interaction data (Supplementary Figure S1). Interestingly, earlier studies revealed that hsa-miR-195-5p could enhance the radiosensitivity of breast cancer cells by repressing the expression of *BCL2* (43). Consequently, it is feasible to discover more potential biomarkers of radiation response by utilizing both information including the associations between factor and cancer in dbCRAF and corresponding interactions between factor and target gene.

This work also has several limitations that require further exploration. On the one hand, the current database lacks information on radiation toxicity, the change of radiosensitivity, treatment options and machines. It is necessary to explore and include this additional information from relevant literature or databases in the future to enhance the understanding of radiation response. On the other hand, we observed that more than half of the associations are linked to head and neck neoplasms, lung neoplasms and breast neoplasms. This disproportionate distribution may introduce bias in the database and the biological information it contains. To address this issue, we are considering implementing mechanisms such as weighting, where cancer types with fewer associations are given greater weight when inferring potential associations between genes and radiation response in cancer. Furthermore, it is crucial to supplement associations of cancer types with limited data by incorporating information from additional sources like Web of Science and Scopus. These efforts will contribute to a more comprehensive representation of radiation response and help mitigate potential bias across various types of cancer.

In conclusion, with the collated data and the implementation of four online analysis tools, dbCRAF is not only an integrated resource, but also a web server for mining the potential molecular mechanisms of radiation response. The data and the functions of dbCRAF may be provide new perspectives on the development of RT therapeutics for human cancer.

## Supplementary Material

zcae008_supplemental_file

## Data Availability

No new data were generated or analysed in support of this research.

## References

[1] Sung H. , Ferlay J., Siegel R.L., Laversanne M., Soerjomataram I., Jemal A., Bray F. Global cancer statistics 2020: GLOBOCAN estimates of incidence and mortality worldwide for 36 cancers in 185 countries. CA Cancer J Clin. 2021; 71:209–249. 33538338 10.3322/caac.21660

[2] Bray F. , Laversanne M., Weiderpass E., Soerjomataram I. The ever-increasing importance of cancer as a leading cause of premature death worldwide. Cancer-Am Cancer Soc. 2021; 127:3029–3030. 10.1002/cncr.33587 34086348

[3] Wang K. , Tepper J.E. Radiation therapy-associated toxicity: etiology, management, and prevention. CA Cancer J Clin. 2021; 71:437–454. 34255347 10.3322/caac.21689

[4] Bentzen S.M. Preventing or reducing late side effects of radiation therapy: radiobiology meets molecular pathology. Nat. Rev. Cancer. 2006; 6:702–713. 16929324 10.1038/nrc1950

[5] Quon H. , McNutt T., Lee J., Bowers M., Jiang W., Lakshminarayanan P., Cheng Z., Han P.J., Hui X., Moore J. et al. Needs and challenges for radiation oncology in the era of precision medicine. Int. J Radiat Oncol. 2019; 103:809–817. 10.1016/j.ijrobp.2018.11.017 30562547

[6] Bernier J. Precision medicine for early breast cancer radiotherapy: opening up new horizons?. Crit. Rev. Oncol. Hemat. 2017; 113:79–82. 10.1016/j.critrevonc.2017.03.015 28427525

[7] Chen H.H.W. , Kuo M.T. Improving radiotherapy in cancer treatment: promises and challenges. Oncotarget. 2017; 8:62742–62758. 28977985 10.18632/oncotarget.18409 PMC5617545

[8] Marks L.B. , Yorke E.D., Jackson A., Ten Haken R.K., Constine L.S., Eisbruch A., Bentzen S.M., Nam J., Deasy J.O. Use of normal tissue complication probability models in the clinic. Int. J Radiat. Oncol. 2010; 76:S10–S19. 10.1016/j.ijrobp.2009.07.1754 PMC4041542 20171502

[9] Galeaz C. , Totis C., Bisio A. Radiation resistance: a matter of transcription factors. Front. Oncol. 2021; 11:662840. 34141616 10.3389/fonc.2021.662840 PMC8204019

[10] Jiao Y. , Cao F., Liu H. Radiation-induced cell death and its mechanisms. Health Phys. 2022; 123:376–386. 36069830 10.1097/HP.0000000000001601 PMC9512240

[11] Xi J.N. , Sun D.H., Chang C., Zhou S.C., Huang Q.H. An omics-to-omics joint knowledge association subtensor model for radiogenomics cross-modal modules from genomics and ultrasonic images of breast cancers. Comput. Biol. Med. 2023; 155:106672. 36805226 10.1016/j.compbiomed.2023.106672

[12] Han Y.Y. , Li F., Xie J., Wang Y., Zhang H. PVT1 mediates cell proliferation, apoptosis and radioresistance in nasopharyngeal carcinoma through regulating miR-515-5p/PIK3CA Axis. Cancer Manag. Res. 2020; 12:10077–10090. 33116864 10.2147/CMAR.S257583 PMC7568593

[13] Wang P. , Sun Y.M., Yang Y., Chen Y.Z., Liu H. Circ_0067835 knockdown enhances the radiosensitivity of colorectal cancer by miR-296-5p/IGF1R axis. Oncotargets Ther. 2021; 14:491–502. 10.2147/OTT.S281011 PMC7822227 33500625

[14] Wen P.B. , Xia J.F., Cao X.B., Chen B., Tao Y.P., Wu L.J., Xu A., Zhao G.P. dbCRSR: a manually curated database for regulation of cancer radiosensitivity. Database-Oxford. 2018; 2018:bay049. 29860480 10.1093/database/bay049 PMC6007213

[15] Principe S. , Zapater-Latorre E., Arribas L., Garcia-Miragall E., Bagan J. Salivary IL-8 as a putative predictive biomarker of radiotherapy response in head and neck cancer patients. Clin. Oral. Invest. 2022; 26:437–448. 10.1007/s00784-021-04017-0 PMC8791883 34251535

[16] Shen J.J. , Yan D.R., Bai L., Geng R.R., Zhao X.L., Li H.J., Dong Y.F., Cao J.P., Tang Z.X., Liu S.B. An 11-gene signature based on treatment responsiveness pedicts radiation therapy survival benefit among breast cancer patients. Front. Oncol. 2022; 11:816053. 35071020 10.3389/fonc.2021.816053 PMC8770413

[17] Song J.H. , Zhang S.M., Sun Y.Y., Gu J.J., Ye Z.Q., Sun X.C., Tang Q.Y. A radioresponse-related lncRNA biomarker signature for risk classification and prognosis prediction in non-small-cell lung cancer. J. Oncol. 2021; 2021:4338838. 34594376 10.1155/2021/4338838 PMC8478572

[18] Zhang C.D. , Yang Y., Chen H.H., Zhang T., Wang Q., Liang Y., Zhang L., Zhou Y. RTPDB: a database providing associations between genetic variation or expression and cancer prognosis with radiotherapy-based treatment. Database. 2018; 2018:bay118. 30376049 10.1093/database/bay118 PMC6206893

[19] Xi J.N. , Yuan X.G., Wang M.H., Li A., Li X.L., Huang Q.H. Inferring subgroup-specific driver genes from heterogeneous cancer samples via subspace learning with subgroup indication. Bioinformatics. 2020; 36:1855–1863. 31626284 10.1093/bioinformatics/btz793

[20] Wei C.H. , Allot A., Leaman R., Lu Z.Y. PubTator central: automated concept annotation for biomedical full text articles. Nucleic Acids Res. 2019; 47:W587–W593. 31114887 10.1093/nar/gkz389 PMC6602571

[21] Tweedie S. , Braschi B., Gray K., Jones T.E.M., Seal R.L., Yates B., Bruford E.A. Genenames.org: the HGNC and VGNC resources in 2021. Nucleic Acids Res. 2021; 49:D939–D946. 33152070 10.1093/nar/gkaa980 PMC7779007

[22] Wishart D.S. , Feunang Y.D., Guo A.C., Lo E.J., Marcu A., Grant J.R., Sajed T., Johnson D., Li C., Sayeeda Z. et al. DrugBank 5.0: a major update to the DrugBank database for 2018. Nucleic Acids Res. 2018; 46:D1074–D1082. 29126136 10.1093/nar/gkx1037 PMC5753335

[23] Lipscomb C.E.J.B.o.t.M.L.A. Medical subject headings (MeSH). Bull. Med. Libr. Assoc. 2000; 88:265–266. 10928714 PMC35238

[24] Safran M. , Rosen N., Twik M., BarShir R., Stein T.I., Dahary D., Fishilevich S., Lancet D Practical Guide to Life Science Databases. 2021; Springer 27–56.

[25] Huang H.Y. , Lin Y.C.D., Cui S.D., Huang Y.X., Tang Y., Xu J.T., Bao J.Y., Li Y.L., Wen J., Zuo H.L. et al. miRTarBase update 2022: an informative resource for experimentally validated miRNA-target interactions. Nucleic Acids Res. 2022; 50:D222–D230. 34850920 10.1093/nar/gkab1079 PMC8728135

[26] Cheng L. , Wang P.P., Tian R., Wang S., Guo Q.H., Luo M., Zhou W.Y., Liu G.Y., Jiang H.J., Jiang Q.H. LncRNA2Target v2.0: a comprehensive database for target genes of lncRNAs in human and mouse. Nucleic Acids Res. 2019; 47:D140–D144. 30380072 10.1093/nar/gky1051 PMC6323902

[27] Dudekulay D.B. , Panda A.C., Grammatikakis I., De S., Abdelmohsen K., Gorospe M. CircInteractome: a web tool for exploring circular RNAs and their interacting proteins and microRNAs. Rna Biol. 2016; 13:34–42. 26669964 10.1080/15476286.2015.1128065 PMC4829301

[28] Liu M. , Wang Q., Shen J., Yang B.B., Ding X.M. Circbank: a comprehensive database for circRNA with standard nomenclature. Rna Biol. 2019; 16:899–905. 31023147 10.1080/15476286.2019.1600395 PMC6546381

[29] Whirl-Carrillo M. , Huddart R., Gong L., Sangkuhl K., Thorn C.F., Whaley R., Klein T.E. An evidence-based framework for evaluating pharmacogenomics knowledge for personalized medicine. Clin. Pharmacol. Ther. 2021; 110:563–572. 34216021 10.1002/cpt.2350 PMC8457105

[30] Subramanian A. , Narayan R., Corsello S.M., Peck D.D., Natoli T.E., Lu X.D., Gould J., Davis J.F., Tubelli A.A., Asiedu J.K. et al. A next generation connectivity map: L1000 platform and the first 1,000,000 profiles. Cell. 2017; 171:1437–1452. 29195078 10.1016/j.cell.2017.10.049 PMC5990023

[31] Szklarczyk D. , Gable A.L., Nastou K.C., Lyon D., Kirsch R., Pyysalo S., Doncheva N.T., Legeay M., Fang T., Bork P. The STRING database in 2021: customizable protein–protein networks, and functional characterization of user-uploaded gene/measurement sets. Nucleic Acids Res. 2021; 49:D605–D612. 33237311 10.1093/nar/gkaa1074 PMC7779004

[32] Kanehisa M. , Goto S. KEGG: Kyoto encyclopedia of genes and genomes. Nucleic Acids Res. 2000; 28:27–30. 10592173 10.1093/nar/28.1.27 PMC102409

[33] Tomczak K. , Czerwińska P., Wiznerowicz M. Review The Cancer Genome Atlas (TCGA): an immeasurable source of knowledge. Contemp Oncol. 2015; 2015:68–77. 10.5114/wo.2014.47136 PMC4322527 25691825

[34] Chow L.Q. Head and neck cancer. N. Engl. J Med. 2020; 382:60–72. 31893516 10.1056/NEJMra1715715

[35] Lemjabbar-Alaoui H. , Hassan O.U., Yang Y.W., Buchanan P. Lung cancer: biology and treatment options. Bba-Rev Cancer. 2015; 1856:189–210. 10.1016/j.bbcan.2015.08.002 PMC4663145 26297204

[36] Al-Mahmood S. , Sapiezynski J., Garbuzenko O.B., Minko T. Metastatic and triple-negative breast cancer: challenges and treatment options. Drug Deliv. Transl. Re. 2018; 8:1483–1507. 10.1007/s13346-018-0551-3 PMC6133085 29978332

[37] Ebahimzadeh K. , Shoorei H., Mousavinejad S.A., Anamag F.T., Dinger M.E., Taheri M., Ghafouri-Fard S. Emerging role of non-coding RNAs in response of cancer cells to radiotherapy. Pathol. Res. Pract. 2021; 218:153327. 33422780 10.1016/j.prp.2020.153327

[38] Cui C.C. , Yang J.B., Li X., Liu D.L., Fu L.W., Wang X.W. Functions and mechanisms of circular RNAs in cancer radiotherapy and chemotherapy resistance. Mol. Cancer. 2020; 19:58. 32171304 10.1186/s12943-020-01180-y PMC7071709

[39] Grimes D.R. Limitations of the radiosensitivity index as a direct prognostic marker. Lancet Oncol. 2022; 23:1352–1353. 36328006 10.1016/S1470-2045(22)00553-8

[40] Sondka Z. , Bamford S., Cole C.G., Ward S.A., Dunham I., Forbes S.A. The COSMIC Cancer Gene Census: describing genetic dysfunction across all human cancers. Nat. Rev. Cancer. 2018; 18:696–705. 30293088 10.1038/s41568-018-0060-1 PMC6450507

[41] Zhou Z.H. , Chen G., Deng C., Tang J.M., Xie L., Zhou H.Y., Ye X., Zhang D.K., Shi R.Q., Tian D. et al. TCF19 contributes to cell proliferation of non-small cell lung cancer by inhibiting FOXO1. Cell Biol. Int. 2019; 43:1416–1424. 31141247 10.1002/cbin.11189

[42] Gong C. , Li W., Wu J., Li Y.Y., Ma Y., Tang L.W. AKBA inhibits radiotherapy resistance in lung cancer by inhibiting maspin methylation and regulating the AKT/FOXO1/p21 axis. J. Radiat. Res. 2022; 64:33–43. 10.1093/jrr/rrac064 PMC9855320 36300343

[43] Zhu J. , Ye Q., Chang L., Xiong W., He Q., Li W. Upregulation of miR-195 enhances the radiosensitivity of breast cancer cells through the inhibition of BCL-2. Int. J. Clin. Exp. Med. 2015; 8:9142. 26309570 PMC4538095

