# Supplementary material for: dbCRAF: a curated knowledgebase for regulation of radiation response in human cancer
Source: NAR Cancer. 2024 Feb 24;6(1):zcae008. doi: 10.1093/narcan/zcae008 (PMC10894039; doi:10.1093/narcan/zcae008)
Supplement: zcae008_supplemental_file [file zcae008_supplemental_file.pdf]

Supplementary Figure

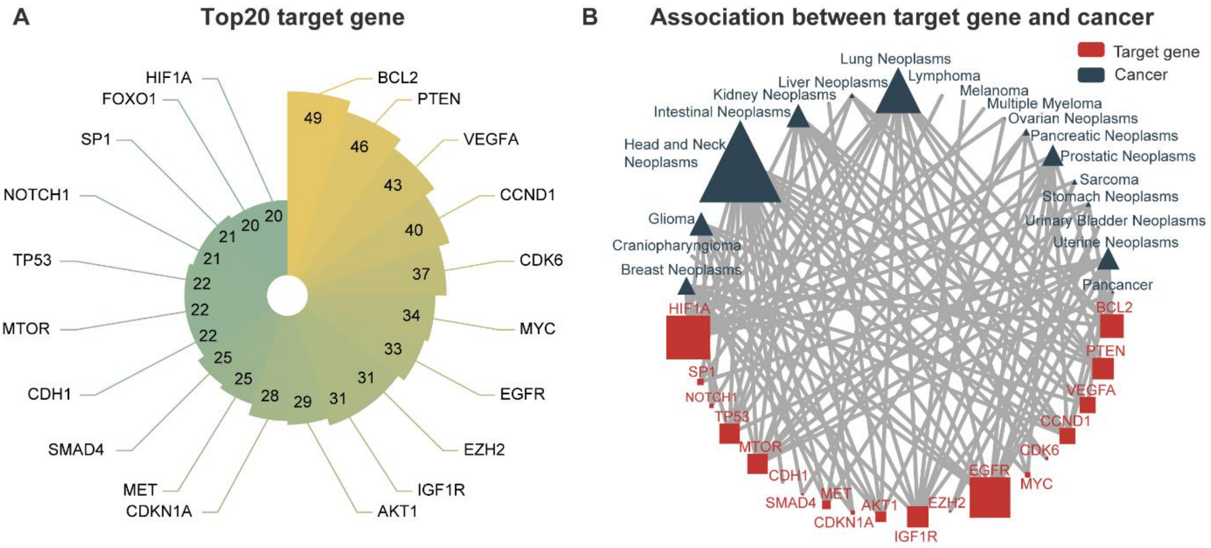

Supplementary Figure S1. Statistics of target genes. (A) Statistic of top20 target gene. **(B)** Association data involving target genes recorded in dbCRAF.

Supplementary Table

Supplementary Table S1. Search strategy.

Search	Query	Text availability	Publication date	Results
	Search (#1) AND #4	[Title/Abstract]	January 2000-February 2022	6,278
	Search (#2) AND #4			4,778
	Search (#1) OR (#2) OR (#3) AND (#4) AND (#5)	[Title]		4,546
#1	Search ((radiosensitivity) OR (radio-sensitivity) OR (radiation sensitivity))			
#2	Search ((radioresistance) OR (radio-resistance) OR (radiation resistance))			
#3	Search ((radiotherapy) OR (radio-therapy) OR (radiation therapy) OR (radioresponse) OR (radio-response) OR (radiation response) OR (radiation oncology) OR (radiation treatment))			
#4	Search ((tumor) OR (cancer) OR (carcinoma)) OR (blastoma) OR (neoplasm))			
#5	Search ((predict) OR (prediction) OR (prognostic) OR (prognosis) OR (analysis) OR (analyze) OR (investigation) OR (model) OR (signature) OR (biomarker))			

Supplementary Table S2. Statistic of interaction data.

Factor	Associated database	Data type	Number (factor-target)
miRNA	miRTarBase	miRNA-target interaction	5,136 (184-2,205)
lncRNA	LncRNA2Target	LncRNA-target interaction	1,001 (54-500)
circRNA	CircInteractome, circbank	CircRNA-target interaction	152 (13-94)
Radiosensitizer	DrugBank, PharmGKB, CMap	Drug-target interaction	936 (197-593)

Supplementary Table S3. The details of 137 genes involved in common four KEGG pathways.

Factor	Gene ID	Species	KEGG pathway
CAMK2G	818	H. sapiens	hsa05022[a], hsa05200[b], hsa04657[c], hsa05205[d]
CHUK	1147	H. sapiens	hsa05022, hsa05200, hsa04657, hsa05205
HRAS	3265	H. sapiens	hsa05022, hsa05200, hsa04657, hsa05205
MAPK1	5594	H. sapiens	hsa05022, hsa05200, hsa04657, hsa05205
PRKCB	5579	H. sapiens	hsa05022, hsa05200, hsa04657, hsa05205
RAC2	5880	H. sapiens	hsa05022, hsa05200, hsa04657, hsa05205
FZD2	2535	H. sapiens	hsa05022, hsa05200, hsa05205
ITPR1	3708	H. sapiens	hsa05022, hsa04657, hsa05205
MAPK13	5603	H. sapiens	hsa05022, hsa04657, hsa05205
TNFRSF1A	7132	H. sapiens	hsa05022, hsa04657, hsa05205
WNT11	7481	H. sapiens	hsa05022, hsa05200, hsa05205
WNT5A	7474	H. sapiens	hsa05022, hsa05200, hsa05205
FOS	2353	H. sapiens	hsa05200, hsa04657, hsa05205

GNA11	2767	H. sapiens	hsa05200, hsa04657, hsa05205
GSTM1	2944	H. sapiens	hsa05200, hsa04657, hsa05205
JAG1	182	H. sapiens	hsa05200, hsa04657, hsa05205
PFN2	5217	H. sapiens	hsa05022, hsa04657
SQSTM1	8878	H. sapiens	hsa05022, hsa04657
IL1B	3553	H. sapiens	hsa05022, hsa04657
DDIT3	1649	H. sapiens	hsa05022, hsa04657
MAP2K6	5608	H. sapiens	hsa05022, hsa04657
PPID	5481	H. sapiens	hsa05022, hsa04657
RPS27A	6233	H. sapiens	hsa05022, hsa04657
TANK	10010	H. sapiens	hsa05022, hsa04657
TNFRSF1B	7133	H. sapiens	hsa05022, hsa04657
TUBB2A	7280	H. sapiens	hsa05022, hsa04657
VDAC1	7416	H. sapiens	hsa05022, hsa04657
ABL1	25	H. sapiens	hsa05200, hsa04657
CCNA2	890	H. sapiens	hsa05200, hsa04657
CCND2	894	H. sapiens	hsa05200, hsa05205
CTBP2	1488	H. sapiens	hsa05200, hsa05205
FGF19	9965	H. sapiens	hsa05200, hsa05205
IFNG	3458	H. sapiens	hsa05200, hsa04657
IGF2	3481	H. sapiens	hsa05200, hsa05205
ITGA2	3673	H. sapiens	hsa05200, hsa05205
JAG2	3714	H. sapiens	hsa05200, hsa05205
MMP1	4312	H. sapiens	hsa05200, hsa04657

RASSF1	11186	H. sapiens	hsa05200, hsa05205
TGFA	7039	H. sapiens	hsa05200, hsa05205
TRAF6	7189	H. sapiens	hsa05200, hsa04657
HCLS1	3059	H. sapiens	hsa04657, hsa05205
ACVR2B	93	H. sapiens	hsa04657, hsa05205
FLNA	2316	H. sapiens	hsa04657, hsa05205
NFATC1	4772	H. sapiens	hsa04657, hsa05205
RPS6KA3	6197	H. sapiens	hsa04657, hsa05205
VAV3	10451	H. sapiens	hsa04657, hsa05205
SNCA	6622	H. sapiens	hsa05022
ADRM1	11047	H. sapiens	hsa05022
ANG	283	H. sapiens	hsa05022
ATP5PD	10476	H. sapiens	hsa05022
CHRM3	1131	H. sapiens	hsa05022
COX7B	1349	H. sapiens	hsa05022
GPX2	2877	H. sapiens	hsa05022
MME	4311	H. sapiens	hsa05022
OPTN	10133	H. sapiens	hsa05022
PSMC2	5701	H. sapiens	hsa05022
PSMD13	5719	H. sapiens	hsa05022
SDHD	6392	H. sapiens	hsa05022
SEPTIN5	5413	H. sapiens	hsa05022
SLC39A14	23516	H. sapiens	hsa05022
SOD1	6647	H. sapiens	hsa05022

TRAP1	10131	H. sapiens	hsa05022
UBA7	7318	H. sapiens	hsa05022
ZFYVE1	53349	H. sapiens	hsa05022
EGLN1	54583	H. sapiens	hsa05200
EPAS1	2034	H. sapiens	hsa05200
LAMB1	3912	H. sapiens	hsa05200
COL4A1	1282	H. sapiens	hsa05200
CUL2	8453	H. sapiens	hsa05200
GNG11	2791	H. sapiens	hsa05200
GNG12	55970	H. sapiens	hsa05200
IL7R	3575	H. sapiens	hsa05200
LAMC2	3918	H. sapiens	hsa05200
PLD1	5337	H. sapiens	hsa05200
ACTN1	87	H. sapiens	hsa04657
CAPNS1	826	H. sapiens	hsa04657
PTPRC	5788	H. sapiens	hsa04657
SUMO1	7341	H. sapiens	hsa04657
WAS	7454	H. sapiens	hsa04657
TJP1	7082	H. sapiens	hsa04657
ARPC1B	10095	H. sapiens	hsa04657
BATF	10538	H. sapiens	hsa04657
BNIP3	664	H. sapiens	hsa04657
BTK	695	H. sapiens	hsa04657
CCL20	6364	H. sapiens	hsa04657

CCL3	6348	H. sapiens	hsa04657
CLDN1	9076	H. sapiens	hsa04657
CLDN4	1364	H. sapiens	hsa04657
CLDN6	9074	H. sapiens	hsa04657
CTLA4	1493	H. sapiens	hsa04657
CXCL10	3627	H. sapiens	hsa04657
CXCL11	6373	H. sapiens	hsa04657
CXCL5	6374	H. sapiens	hsa04657
FOSL2	2355	H. sapiens	hsa04657
HSPA4	3308	H. sapiens	hsa04657
ICAM1	3383	H. sapiens	hsa04657
IFIT1	3434	H. sapiens	hsa04657
ISG15	9636	H. sapiens	hsa04657
ITGAM	3684	H. sapiens	hsa04657
JUND	3727	H. sapiens	hsa04657
MAP3K8	1326	H. sapiens	hsa04657
MAPK6	5597	H. sapiens	hsa04657
MAVS	57506	H. sapiens	hsa04657
MIB1	57534	H. sapiens	hsa04657
MMP13	4322	H. sapiens	hsa04657
MMP3	4314	H. sapiens	hsa04657
MUC5B	727897	H. sapiens	hsa04657
MX1	4599	H. sapiens	hsa04657
MYO1B	4430	H. sapiens	hsa04657

MYO5C	55930	H. sapiens	hsa04657
NOS3	4846	H. sapiens	hsa04657
P2RX7	5027	H. sapiens	hsa04657
PAK2	5062	H. sapiens	hsa04657
PAK4	10298	H. sapiens	hsa04657
PDCD1	5133	H. sapiens	hsa04657
RPSA	3921	H. sapiens	hsa04657
SIRPA	140885	H. sapiens	hsa04657
SYK	6850	H. sapiens	hsa04657
TXNIP	10628	H. sapiens	hsa04657
ITGB5	3693	H. sapiens	hsa05205
CD63	967	H. sapiens	hsa05205
COL1A2	1278	H. sapiens	hsa05205
LUM	4060	H. sapiens	hsa05205
AFP	174	H. sapiens	hsa05205
CCN2	1490	H. sapiens	hsa05205
CYP21A2	1589	H. sapiens	hsa05205
DCN	1634	H. sapiens	hsa05205
HSPB2	3316	H. sapiens	hsa05205
ID3	3399	H. sapiens	hsa05205
KLF4	9314	H. sapiens	hsa05205
LATS1	9113	H. sapiens	hsa05205
PAX6	5080	H. sapiens	hsa05205
PPP1R12A	4659	H. sapiens	hsa05205

SMARCA2	6595	H. sapiens	hsa05205
SMARCC1	6599	H. sapiens	hsa05205
SOX17	64321	H. sapiens	hsa05205
TIMP3	7078	H. sapiens	hsa05205

[a]hsa05022: Pathways of neurodegeneration. [b]hsa05200: Pathways in cancer. [c]hsa04657: IL-17 signaling pathway. [d]hsa05205: Proteoglycans in cancer.
